# Secondary right atrial thrombosis in three dogs: Antithrombotics therapy and echocardiographic follow‐up

**DOI:** 10.1002/vms3.1210

**Published:** 2023-07-25

**Authors:** Tomohiko Yoshida, Akiko Uemura, Ryou Tanaka, Ahmed Farag, Ahmed S. Mandour, Lina Hamabe, Kotaro Matsumoto

**Affiliations:** ^1^ Department of Clinical Veterinary Medicine Obihiro University of Agriculture and Veterinary Medicine Hokkaido Japan; ^2^ Department of Veterinary Surgery Tokyo University of Agriculture and Technology Tokyo Japan; ^3^ Faculty of Veterinary Medicine, Department of Surgery Anesthesiology, and Radiology, Zagazig University Zagazig Egypt; ^4^ Faculty of Veterinary Medicine, Department of Animal Medicine (Internal Medicine) Suez Canal University Ismailia Egypt

**Keywords:** canine, clopidogrel, echocardiography, intracardiac thrombosis, rivaroxaban

## Abstract

Three dogs were diagnosed with right atrial thrombosis, thought to be secondary to systemic diseases. Specifically, two cases had hyperadrenocorticism and one case was diagnosed with pancreatitis with acute renal injury. In all cases, the thrombi were found within the right atrium, necessitating a differentiation from cardiac neoplasia. In all three cases, the structures assumed to be thrombi had irregular margins with interspersed hypoechoic regions, which were later confirmed as thrombi based on the responsiveness to therapy. All three cases were prescribed with the combination of clopidogrel and rivaroxaban.The thrombi gradually disappeared after initiation of the combination therapy. Complete resolution of right atrial thrombosis was noted in each dog treated with clopidogrel and rivaroxaban. This combination therapy appears to be safe and well tolerated. Diligent observation of the echocardiographic findings and clinical course allows the diagnosis of thrombosis.

## Graphical abstract

Three dogs were diagnosed with right atrial thrombosis, thought to be secondary to systemic diseases. All three cases were prescribed with the combination of clopidogrel and rivaroxaban. The thrombi gradually disappeared after initiation of the combination therapy. (A thrombus gradually disappeared in Case 3; A→B→C). The combination of clopidogrel and rivaroxaban appears to be well tolerated and effective in dogs with intracardiac thrombosis.

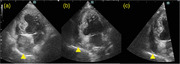

## 1 INTRODUCTION

It is well known that atrial fibrillation causes intracardiac thrombosis in humans (Lim et al., 2013; Petersen et al., 1990). In dogs, the incidence of cardiac‐related intracardiac thrombosis is lower than in cats; incidents of intracardiac thrombosis have been reported after pacemaker implantation, mitral valvuloplasty; and secondary to other underlying conditions (Matsuura et al., 2022; Mulz et al., 2010). While immune‐mediated haemolytic anaemia, sepsis, protein‐losing nephropathy/enteropathy, malignant tumours and hyperadrenocorticism have been proposed as predisposing factors of thromboembolism in dogs, broad‐scale studies on intracardiac thrombosis are limited, and information concerning the progression, treatment, and outcomes of the disease remains largely unknown (Goggs et al., 2019). In cats, cardiac‐related thrombosis is associated with poor prognosis, on the other hand, few reports on the prognosis of canine intracardiac thrombosis exist (Borgeat et al., 2014). Furthermore, reports on the progression of the intracardiac thrombus with regular monitoring after treatment have been limited. In human medicine, the combination of rivaroxaban and clopidogrel has been used for the treatment of thrombosis, and similarly it has recently attracted attention in small animal practice (Goggs et al., 2019). A combination of these two medications has been reported to improve the prognosis of feline myocardiopathy‐related thrombosis. However, there are limited number of studies on its usefulness in intracardiac thrombosis in dogs. This case report describes the gradual resolution of intracardiac blood clots in three dogs managed with the combination of clopidogrel and rivaroxaban.

## 2 CASE SUMMARY

### 2.1 Case 1

**FIGURE 1:**
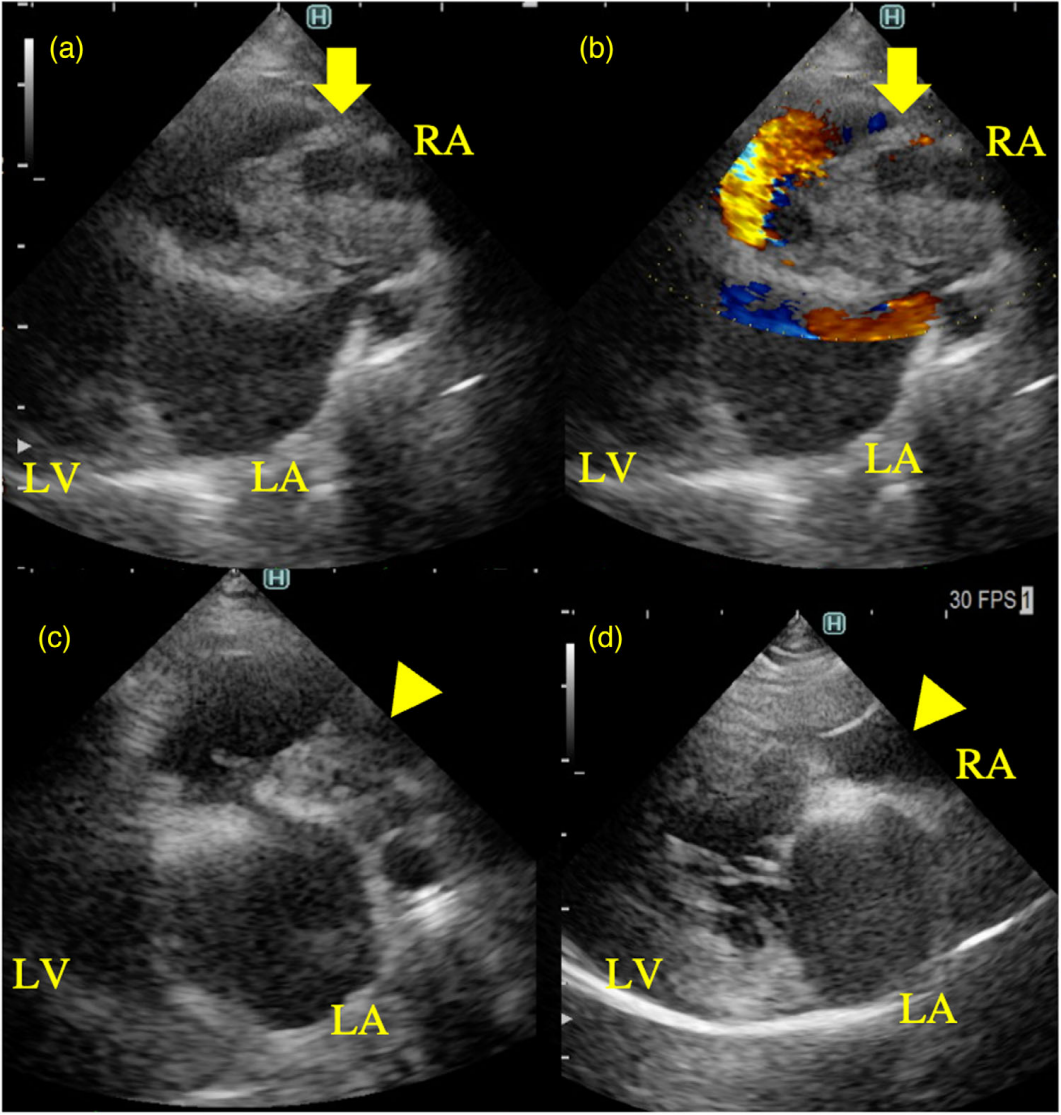
Echocardiographic images taken before (a, b) and after (c, d) the antithrombotic therapy in Case 1 diagnosed with hyperadrenocorticism and pancreatitis. a: Right parasternal long‐axis view before antithrombotic therapy, showing a thrombus (indicated by the yellow arrow) occupying the right atrium. b: Right parasternal long‐axis view with colour Doppler imaging before antithrombotic therapy, demonstrating the thrombus obstructing the blood flow. c: Right parasternal long‐axis view after antithrombotic therapy on Day 40, illustrating partial resolution of the thrombus after treatment indicated by the yellow arrowhead . d: Right parasternal long‐axis view after antithrombotic therapy on Day 68, which the thrombus in the right atrium had disappeared shown by the yellow arrowhead. RA: right atrium, RV: right ventricle, LA: left atrium, LV: left ventricle.

An 11‐year‐old castrated male chihuahua was presented with anorexia and dyspnoea. The patient was reported to be in good general condition with normal appetite until the day before the presentation which dyspnoea started suddenly. Clinical signs of polyuria and polydipsia had also been noted 3 months earlier, and abdominal distension and generalised alopecia were noted during the physical examination. Haematology and biochemical results revealed elevated WBC, ALT, AST, ALP, GGT, lipase and CRP (Table 1: Pretreatment, Case 1). Thoracic radiograph suggested an enlarged right heart (VHS: vertebral heart size, 11.0 v) and unstructured interstitial and alveolar lung patterns in all lung lobes, especially in the right middle lobe. Abdominal and cardiac ultrasound examinations were performed using a ARIETTA 850 with 5‐MHz and 10‐MHz transducer probes (FUJIFILM) while receiving supplemental oxygen. Echocardiography revealed a hyperechoic structure inside the right atrium (Figure 1A), which was obstructing the blood flow (Figure 1B). The structure assumed an irregular margin and was interspersed with hypoechoic regions (Figure 1, A and B, yellow arrow). The evidence of abdominal and pleural effusion, distension of the pulmonary artery and tricuspid regurgitation were not detected. No other cardiovascular diseases were observed. During ultrasound examination of the abdomen, enlarged adrenal glands (left adrenal gland 6.6 mm, right adrenal gland 7.0 mm), hyperechoic change in the peripancreatic region, and a corrugated pattern of the intestine were noted. D‐dimer had a high reading of 10.1 μg/mL, while values of PT, APTT and fibrinogen were normal.

**TABLE 1:** Haematological and biochemical analysis in the three dogs with intracardiac thrombosis.

	Before treatment				After treatment			
Variables	Unit	Case 1	Case 2	Case 3	Case 1	Case 2	Case 3	Reference range (Chang et al., 2016; Dixon‐Jimenez et al., 2013; Han & Kim, 2022; Lilliehöök & Tvedten, 2009)
WBC	10^2^/μL	208	188	317	107	88	17.6	60–170
HGB	g/dL	14.5	15.7	8.5	15.1	16.1	12.5	12–18
HCT	%	41	42.9	31.1	42.1	44	35.1	37–55
PLT	10^3^/μL	22	44.4	16.3	22.6	26.3	20.7	20–50
PT	s	7	7.2	7.5	7.1	7.3	7.7	6.0–8.6
APTT	s	24	21.2	20.6	25.3	24.2	26	13.1–26.9
Fib	mg/dL	108	202	112	303	150	101	88–336
D‐dimer	μg/mL	10.1	11.28	6.1	1.1	0.5	1.9	<2.0
TP	g/dL	6.5	6.1	6.8	7.0	6.8	7.0	5.8–7.5
Alb	g/dL	2.9	2.3	3.2	3.6	2.9	3.4	2.9–3.8
GLU	mg/dL	99	101	98	107	121	98	75‐128
GGT	IU/L	33	12	7	2	3	5	0–7
BUN	mg/dL	28.3	52	20.3	17.2	21	20.6	9.8–30.6
Cre	mg/dL	1.43	2.03	0.71	1.1	0.52	0.51	0.26–1.6
ALT	IU/L	203	303	62	88	63	77	14–76
AST	IU/L	300	103	66	97	61	57	10–70
ALP	IU/L	1112	605	34	654	110	90	12–93
Na	mEq/L	145	149	152	148	153	146	144–152
K	mEq/L	3.2	4.1	4.0	4.6	4.2	4.0	3.5–4.5
Cl	mg/dL	114	104	107	104	105	105	105–119
IP	mg/dL	5.6	3.6	3.0	4.3	4.2	3.7	2.0–4.6
Ca	mg/dL	10.1	9.5	10.5	11.0	11.5	11.1	9.4–11.2
LIPA	IU/L	450	617	315	102	123	99	10–160
CRP	mg/dL	12	14.7	0.1	0.7	0.9	0.5	0.0–0.99

Abbreviations: WBC, white blood cells; RBC, red blood cells, HGB, haemoglobin; HCT, haematocrit; PLT, platelet count; PT, prothrombin time; APTT, activated prothrombin time; Fib, fibrinogen; TP, total protein; Alb, albumin; GLU, glucose; GGT, gamma‐glutamyl transferase; BUN, blood urea nitrogen; Cre, creatinine; ALT, alanine aminotransferase; AST; aspartate transferase; ALP, alkaline phosphatase; Na, sodium; K; potassium; Cl, chloride; IP, inorganic phosphorus; Ca, calcium; LIPA, lipase; CRP, C‐reactive protein.

Based on the above findings, intracardiac thrombosis secondary to hyperadrenocorticism and pancreatitis was suspected. Dyspnoea improved after 3 h of oxygen therapy. Clopidogrel (2 mg/kg, PO, q24h; Clopidogrel Sulfate, Sanofi K.K.) was prescribed for the treatment of thrombosis. On Day 14 (after the first visit, omitted below), while clinical signs improved, there was no change in the thrombus size. Hence, rivaroxaban (1 mg/kg, PO, q24h; Xarelto, Bayer Yakuhin, Ltd.) was additionally prescribed. Moreover, trilostane (3.0 mg/kg, PO, q24h; Adrestane,Kyoritsu Seiyaku Corporation) was started based on the increased cortisol concentration (ACTH stimulation test: precortisol 8.3 μg/dL, postcortisol >50 μg/dL). On Day 40, an echocardiography revealed a decrease in the size of the intracardiac structure (Figure 1C), which supported the diagnosis of the intracardiac structure as a thrombus. On Day 68, the thrombus in the right atrium disappeared (Figure 1D). The elevated D‐dimer concentration declined below the reference range, and PT, APTT and fibrinogen levels remained normal (Table 1: Posttreatment, Case 1). After the resolution of the thrombus, the patient continues to be treated for hyperadrenocorticism and prevented the relapse of thrombosis.

### 2.2 Case 2

**FIGURE 2:**
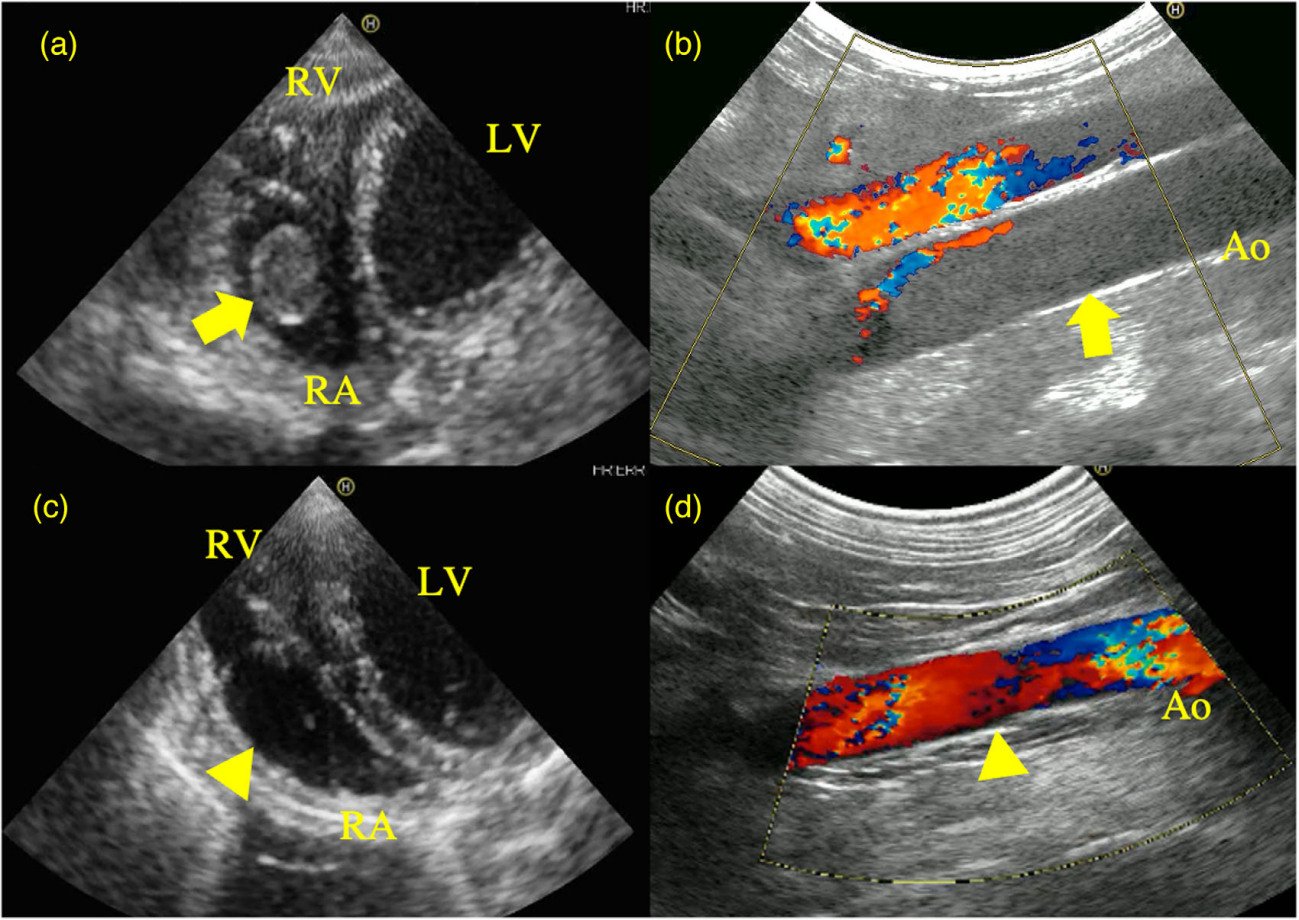
Echocardiographic and abdominal ultrasound images before (a, b) and after (c, d) antithrombotic therapy in Case 2 diagnosed with acute kidney injury and pancreatitis. a: Left parasternal apical four‐chamber view before antithrombotic therapy. b: Two‐dimensional longitudinal ultrasound with colour Doppler imaging of the aorta before antithrombotic therapy. Yellow arrows indicate a hyperechoic mass representing a thrombus within (a) the right atrium a and (b) aortic lumen. c: Left parasternal apical four‐chamber view after antithrombotic therapy. d: Two‐dimensional longitudinal ultrasound with colour Doppler imaging of the aorta after antithrombotic therapy. The thrombus in the right atrium and aortic lumen had disappeared indicated by the yellow arrowheads. RA: right atrium, RV: right ventricle, LV: left ventricle.

A 14‐year‐old castrated male miniature Dachshund was presented for anorexia and dyspnoea. This dog was unable to stand up on its own . On physical examination, the paw pads of the hindlimbs demonstrated superficial pain sensation and were cold on palpation. Pulse was lost on the left femoral artery and attenuated on the right. Proprioceptive responses were not noted in both hindlimbs. The respiratory rate was 80 per minute. Blood test revealed elevated WBC, ALT, AST, ALP, BUN, creatinine, lipase and CRP (Table 1: Pretreatment, Case 2) and a mild decrease in albumin concentration. A mild increase in blood pressure was also recognised; systolic, diastolic and mean readings were 171, 114 and 134 mmHg, respectively, with a pulse rate of 126 bpm. Thoracic radiograph suggested a mild cardiomegaly (VHS: vertebral heart size, 11.1v) with no abnormality detected in the lung field. Echocardiography revealed an isolated hyperechoic structure within the right atrium in the left parasternal four‐chamber view (Figure 2A). The structure had internal hypoechoic regions and an irregular margin. No abdominal or pleural effusion was detected on ultrasound examination, nor was distension of the pulmonary artery or tricuspid regurgitation noted. Abdominal ultrasound revealed increased echogenicity of the peripancreatic region. The hyperechoic structure was also found in the aortic lumen at the level of aortic bifurcation, obstructing blood flow (Figure 2B, arrow). The D‐dimer concentration was elevated with normal readings of PT, APTT and fibrinogen (Table 1: Pretreatment, Case 2).

Based on the above findings, pancreatitis, renal insufficiency and intracardiac/intraaortic thrombosis were suspected. Low‐molecular‐weight heparin (100 IU/kg, SC, TID; Fragmin, Kissei Pharmaceutical Company) was initiated as antithrombotic therapy until the respiratory status improved on Day 2, which then was switched to clopidogrel (2 mg/kg, PO, q24h). By Day 14, there was an improvement in the clinical signs and the results of blood test, albeit no change in the size of the intracardiac thrombus. Therefore, rivaroxaban (1 mg/kg, PO, q24h) was additionally prescribed. On Day 35, a reduction in the size of intracardiac and intraaortic structures was noted, which supported the diagnosis of intracardiac/intraaortic structure as thrombi. On Day 65, the intracardiac and intraaortic thrombus were no longer visible on ultrasound (Figure 2C and D). The D‐dimer value improved while PT, APTT and fibrinogen levels remained normal (Table 1: Posttreatment, Case 2). At the time of writing, the dog continues to receive prophylactic antithrombotic therapy.

### 2.3 Case 3

**FIGURE 3:**
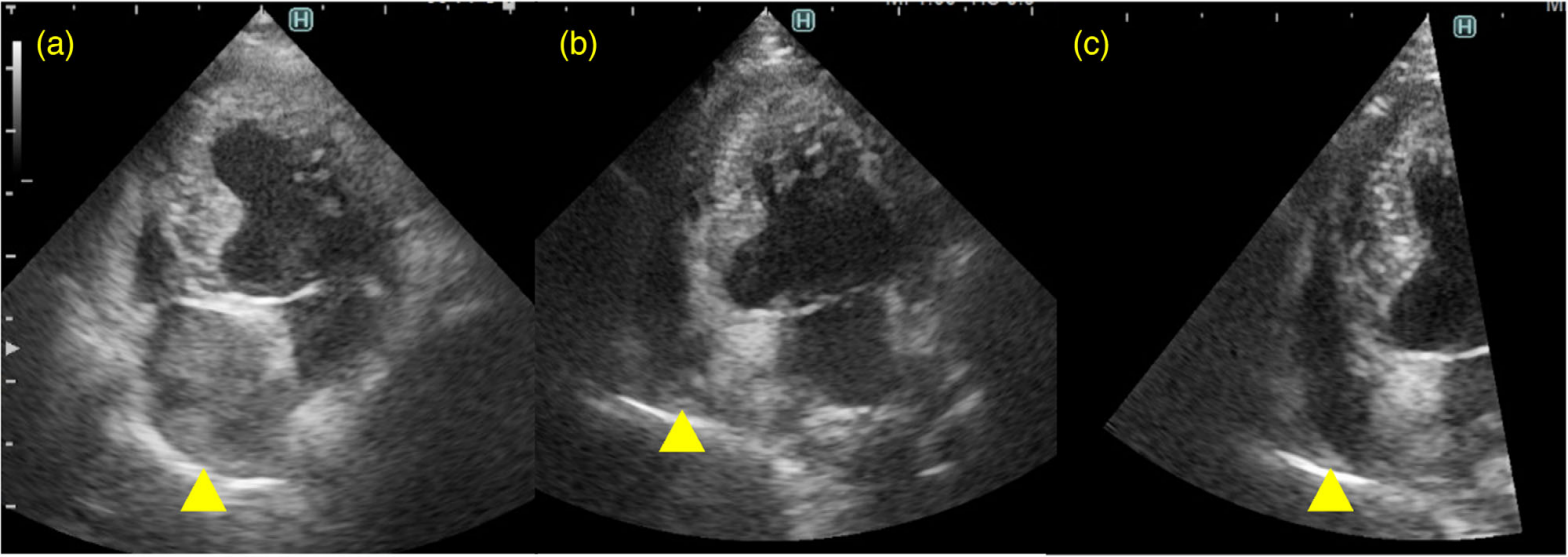
Echocardiographic images taken at left parasternal apical four‐chamber view in Case 3 with diagnosed with hyperadrenocorticism and pancreatitis on Day 1 (a), Day 14 (b), and Day 93 (c). Yellow arrowheads indicate the hyperechoic mass representing a thrombus within the right atrium. Gradual disappearance of the thrombus can be observed. The scan lines were narrowed down to increase the resolution of the image in c.

A 10‐year‐old male miniature Dachshund was presented for lethargy, anorexia and dyspnoea. The dog had been diagnosed with hyperadrenocorticism and was prescribed trilostane (3.5 mg/kg, PO, q24h) at another veterinary clinic. On physical examination, no heart murmur was auscultated, but an increased respiratory rate and fine crackles were noted. Thoracic radiography indicated right heart enlargement and increased opacity of the entire lung field. Blood test revealed increased WBC, ALT, AST, ALP, lipase and CRP (Table 1: Pretreatment, Case 3). Echocardiography revealed a hyperechoic structure inside the right atrium (Figure 3A). The hyperechoic structure had an irregular margin and scattered hypoechoic regions on the interior. Abdominal ultrasound noted pancreatic enlargement and increased echogenicity of the surrounding mesentery. Additionally, D‐dimer revealed an elevated value of 6.1 μg/mL and normal PT, APTT, and fibrinogen readings (Table 1: Pretreatment, Case 3).

Based on the above findings, intracardiac thrombosis secondary to hyperadrenocorticism and pancreatitis was suspected. The dog was hospitalised, and supplemental oxygen and antithrombotic therapy initiated. Low‐molecular‐weight heparin (100 IU/kg, SC, TID; Fragmin, Kissei Pharmaceutical Company)was administered until an improvement in respiratory status was observed on Day 3 of treatment, which then the low‐molecular‐weight heparin was replaced with clopidogrel (2 mg/kg, PO, q24h) and rivaroxaban (1 mg/kg, PO, q24h). On Day 14, shrinkage of the intracardiac thrombus was noted (Figure 3B), alongside improved WBC, GPT, GOT, ALP, lipase, CRP and D‐dimer values (Table 1: Posttreatment, Case 3). No abnormality in PT, APTT or fibrinogen results was recognised. Diagnosis of the intraatrial structure as a thrombus was made based on the responsiveness to therapy. The dyspnoea was thought to be due to pulmonary thromboembolism; however, a definitive diagnosis could not be made without performing a CT scan. Three months after initial therapy, there was no recognisable intracardiac thrombus on echocardiography and the dog was in a stable condition (Figure 3C). The dog continues to receive antithrombotic combination therapy even after complete resolution of the atrial thrombosis.

## 3 DISCUSSION

This case report describes clinical findings and outcomes of three dogs with intracardiac thrombosis, considered to be secondary to systemic diseases. All dogs were managed with the combination of clopidogrel and rivaroxaban, which resulted in complete resolution of thrombi.

Recently in veterinary medicine, the increased availability of imaging modalities has improved the ability to accurately diagnose thromboses. Based on evidences from literature, antiplatelet drugs such as clopidogrel and aspirin, and anticoagulants such as rivaroxaban and heparin preparations are being used commonly (Blais et al., 2019; Goggs et al., 2019). Thrombosis can be found in various diseases where high mortality has been an issue (Goggs et al., 2019). The correlation between thrombosis and inflammation and the occurrence of multiple organ failures as a consequence of thrombosis necessitate appropriate and prompt treatment (Esmon, 2003; Gando, 2010). In humans, an evidence‐based guideline on thrombosis has been established (Kearon et al., 2012; Kearon et al., 2016). Although guidelines concerning thrombosis in small animal medicine have also been published, there is not enough consensus compared to human medicine (Goggs et al., 2019). As stated in the guidelines, diseases associated with a high risk of thrombosis include immune‐mediated haemolytic anaemia (IMHA), hypoalbuminemia, cardiac diseases, arrhythmia, filariasis, hyperadrenocorticism, sepsis, hepatic diseases, congenital portosystemic shunt, neoplasia and pancreatitis, alongside a history of corticosteroid administration (Goggs et al., 2019). Complications with these diseases lead to a higher risk of thrombosis. Cases 1−3 were all complicated with guideline‐stated diseases with high risk of thrombosis. In cases of conditions with a high risk of thrombosis, formation of thrombosis should be strongly suspected. A thorough systemic screening is considered necessary to search for any evidence of thrombosis.

All three cases in this report had thrombosis in the right atrium, which must be distinguished from intracardiac neoplasia. Few reports exist on the imaging findings of intracardiac thrombosis. On the other hand, some papers exist on the imaging findings of intracardiac neoplasia. Yamamoto et al. (2013) summarised the ultrasonographic findings of cardiac neoplasia. Among the intracardiac neoplasia, those of right atrial origin were most prevalent, with haemangiosarcoma being most commonly diagnosed. However, how neoplasia is differentiated from thrombosis based on imaging characteristics was not mentioned, and most diagnoses were reached after necropsy. All three cases in this report had thrombosis inside the right atrium, which must be distinguished from intracardiac neoplasia. In Case 3, the structure recognised in the right atrium was found to have an irregular margin and scattered hypoechoic regions in the interior. The structure of Case 2 was found floating in the blood flow. Since elevated D‐dimer concentration was recognised in all three cases, the structure inside the right atrium was tentatively diagnosed as thrombi. Moreover, observation of regression and disappearance after treatment allowed a diagnosis of thrombosis. In veterinary medicine, previous reports on intracardiac thrombus exist, although the description of imaging findings is insufficient, making the comparison with the cases in this report difficult (Warman et al., 2006). Moreover, no detailed reports could be found regarding the course after treatment. Although contrast echocardiography has been proposed to distinguish the thrombi from tumours in human medicine, it is difficult to definitively distinguish between these two using plane 2D echocardiography (Mansencal et al., 2009). As a premortem definitive diagnosis of intracardiac thrombosis is achievable based on the history and response to treatment, sequential observation of the structure inside the right atrium and assessment of underlying disease is very important. As noted in the previous report, elevated D‐dimer level is one of the findings suggesting the presence of thrombosis. Although imaging findings of this report are not specific to thrombosis, meticulous examination of the structure inside the right atrium with ultrasound helps diagnosis of intracardiac thrombosis (Epstein et al., 2013; Nelson & Andreasen, 2003).

There are reports on treating canine intracardiac thrombosis using warfarin, clopidogrel and tPA (Blais et al., 2019). Combination therapy of clopidogrel and rivaroxaban for treatment of intrapulmonary arterial thrombosis has been reported, albeit no such report exists on the same combination therapy for treatment of intracardiac thrombosis with chronological evaluation of imaging findings (Tracy et al., 2022). All three cases in this report were treated with the combination of clopidogrel and rivaroxaban, and intracardiac thrombus disappeared within 3 months of treatment. Few reports exist on the use of rivaroxaban in veterinary medicine and there is no consensus on the appropriate dosage and frequency. In contrast, the use of clopidogrel has been reported often, and for such reason, clopidogrel was chosen as the initial treatment. In Case 2, clopidogrel was particularly indicated as an antiplatelet drug due to the coincidental finding of right atrial and arterial thromboses. However, regression of thrombosis was poor with clopidogrel monotherapy, while addition of rivaroxaban resulted in an excellent treatment response where the thrombus disappeared. Rivaroxaban exerts its anticoagulant effect by directly antagonising the factor Xa and has been proven an effective anticoagulant in various pathological conditions in human medicine. Rivaroxaban has also attracted attention in small animal medicine; however, little is known about its usefulness when combined with clopidogrel. In human medicine, previous reports reported that safety and efficacy evaluation of antithrombotic therapy with rivaroxaban and clopidogrel after percutaneous coronary intervention (references). Additionally in cats, combination therapy of clopidogrel and rivaroxaban has been reported to be effective against cardiomyopathy‐related thrombosis with minimal side effects. However, large‐scale studies have not yet been conducted in dogs (Lo et al., 2022). In this report, none of the three dogs developed abnormalities in coagulation or platelet counts after administering combination therapy, suggesting a good treatment tolerability. In addition, the concept of the cell‐based coagulation model, emphasising the presence of a substantial interrelation between platelets and coagulation factors, has recently become widespread (Smith, 2009). Hence, a favourable response may be expected with initiation of the antiplatelet‐anticoagulant combination early in the treatment course (Blais et al., 2019).

There are several limitations in this retrospective case report. The first is that tentative diagnosis was made based simply on the imaging findings, and diagnosis of thrombosis remains challenging. Sequential evaluation and assessment of the treatment response are required to distinguish intracardiac thrombus from tumour. Second, the optimal timing of rivaroxaban inclusion remains unknown. Clopidogrel monotherapy might as well have been effective against thrombosis if continued long enough. As rivaroxaban is an expensive drug and combining it may increase the risk of developing severe bleeding diathesis, where caution is advised when administering it with clopidogrel. Third, although prophylactic antithrombotic therapy is currently being continued in all cases, little evidence exists to support whether the cessation of the therapy is indicated; the optimal time of cessation remains unknown. Further investigation based on a larger study population is needed.

In conclusion, the current case report informs the combination therapy of clopidogrel and rivaroxaban in three dogs with intraatrial thrombosis. Improvement in imaging findings is recognised in all cases with no severe side effects. It is important to differentiate intracardiac thrombosis from cardiac neoplasia. Diagnosis is possible with meticulous repeated assessment by echocardiography.

## AUTHOR CONTRIBUTIONS

T.Y., A.U. and K.M. designed the report and drafted the manuscript. A.F., A.M. and L.H. drafted the manuscript and analysed data. R.T. and K.M. edited the final version and critically reviewed the manuscript.

## CONFLICT OF INTEREST STATEMENT

The authors declare there is no conflict of interest.

## FUNDING

The authors received no specific grant from funding agencies in the public, commercial or not‐for‐profit sectors.

### ETHICS STATEMENT

Ethics approval was not needed because all the information was obtained from clinical diagnosis.

### PEER REVIEW

The peer review history for this article is available at https://publons.com/publon/10.1002/vms3.1210.

## Data Availability

The raw data supporting the conclusions of this article will be made available by the authors, without undue reservation.
